# Effect of Lactoferrin on the Expression Profiles of Long Non-coding RNA during Osteogenic Differentiation of Bone Marrow Mesenchymal Stem Cells

**DOI:** 10.3390/ijms20194834

**Published:** 2019-09-28

**Authors:** Yan Xu, Jing-Jing An, Dina Tabys, Yin-Dan Xie, Tian-Yu Zhao, Hao-Wei Ren, Ning Liu

**Affiliations:** 1 Key Laboratory of Dairy Science, Ministry of Education, Northeast Agricultural University, Harbin 150030, China; xuyan1991521@163.com (Y.X.); anjingloveme@163.com (J.-J.A.); tabysdina@gmail.com (D.T.); xieyindan99@163.com (Y.-D.X.); 13091456816@163.com (T.-Y.Z.); 2 College of Food Science, Northeast Agricultural University, Harbin 150030, China

**Keywords:** lactoferrin, bone marrow mesenchymal stem cells, osteogenic differentiation, lncRNAs, RNA-seq

## Abstract

Lactoferrin (LF) has demonstrated stimulation of osteogenic differentiation of mesenchymal stem cells (MSCs). Long non-coding RNAs (lncRNAs) participate in regulating the osteogenic differentiation processes. However, the impact of LF on lncRNA expression in MSC osteogenic differentiation is poorly understood. Our aim was to investigate the effects of LF on lncRNAs expression profiles, during osteogenic differentiation of rat bone marrow mesenchymal stem cells (rBMSCs), by RNA sequencing. A total number of 1331 putative lncRNAs were identified in rBMSCs during osteogenic differentiation in the study. LF influenced the expression of 120 lncRNAs (differentially expressed lncRNAs [DELs], Fold change > 1.5 or < −1.5; *p* < 0.05) in rBMSCs on day 14 of osteogenic differentiation, consisted of 60 upregulated and 60 down-regulated. Furthermore, the potential functions of DELs were of prediction by searching their target cis- and trans-regulated protein-coding genes. The bioinformatic analysis of DELs target gene revealed that LF led to the disfunction of transforming growth factor beta stimulus (TGF-β) and positive regulation of I-κappa B kinase/NF-κappa B signaling pathway, which may relate to osteogenic differentiation of rBMSCs. Our work is the first profiling of lncRNA in osteogenic differentiation of rBMSCs induced by LF, and provides valuable insights into the potential mechanisms for LF promoting osteogenic activity.

## 1. Introduction

Bone marrow mesenchymal stem cells (BMSCs) are known to be an essential sources of progenitor cells, with multipotent and self-renewal capacity to differentiate into various cell types, including chondrocytes, osteoblasts, and adipocytes [1,2]. The osteogenic differentiation of BMSCs is responsible for bone formation, regeneration, and repair [3]. In recent times, some naturally existing nutrients and bioactive compounds, including fatty acid [4], carotenoids [5,6], flavonoids [7,8] phenolic acids [9], and peptides [10,11] have shown that enhanced osteogenic differentiation of osteoblasts and mesenchymal stem cells (MSCs) have attracted more attention as a source of clinical and functional food applications for improving bone health. Lactoferrin (LF), an iron-binding glycoprotein in the transferrin family, existed in mammalian biological secretory fluids (such as milk, saliva, and other exocrine secretion) [12]. It is reported that LF significantly enhances bone regeneration capacity in a calvarial defect rat model [13] and distraction osteogenesis in rabbit model [14]. LF also conserves bone homeostasis in ovariectomized BALB/c mice [15]. In addition, LF exhibits a promising role in the stimulation of osteogenic differentiation of osteoblasts [16], adipose stem cells [17], and MSCs [18]. Thus LF is a potent anabolic bone formation and growth factor, and has developed as an effector molecule on bone remodeling. However, the mechanisms of LF-promoting osteogenic activity were unclear.

Several studies attempted to investigate mechanisms underlying the osteotropic activity of LF. For example, it was suggested that LF stimulates osteoblast proliferation through binding to low-density lipoprotein receptor-related protein 1 (LRP1) and as a result activates the MAPK-extracellular signal regulated kinase (ERK) 1/2 [19]. The MAPK signaling pathway participates in the promotion of osteoblasts proliferation and osteoblast differentiation by LF [16,20]. Moreover, LF induces osteoblast osteogenic differentiation by binding to TGF-β receptor II followed activation of TGF-β signaling pathway [18,21]. Collectively, these studies indicate that LF promoting osteoblast osteogenic activity is involved in various signaling pathways, but the mechanism of LF in osteogenic differentiation of BMSCs is not fully understood. The long non-coding RNAs (lncRNAs) have been shown to regulate osteogenic differentiation of MSCs [22,23,24]. However, it has not been reported whether LF has had an effect on lncRNA expression during osteogenic of BMSCs, which is important for LF applied in functional food and therapeutic effects in promoting bone remodeling and bone health.

The lncRNAs are a heterogeneous class of long transcripts [>200 nucleotides (nt)] without protein-coding potential [25]. They modulate various biological processes, including cell growth, differentiation, and transcriptional regulation [26,27,28]. The lncRNAs have a special role in the osteogenic differentiation of MSCs at transcriptional, post-transcriptional, and post-translational levels [29,30], such as regulated osteogenic lineage-associated gene transcription via relation to adjacent mRNA, regulation of transcription factors by modulation of histone acetylation status, modulated osteogenic differentiation by playing a role of transcription factors decoys, and modulated osteogenesis by targeting microRNAs. Upregulation of lncRNA MEG3 partly promotes osteogenic differentiation of MSCs by activating bone morphogenetic protein 4 (BMP4) transcription, which is identified as a regulator of cartilage and bone formation [31]. Also, lncRNA KCNQ1OT1 promotes bone morphogenetic protein 2 (BMP2) expression and regulates osteogenic differentiation by sponging miRNA-214 [32]. In another study, the differentiation antagonizing non-protein coding RNA (DANCR) participates and regulates the proliferation and osteogenic differentiation of mesenchymal stem cells, derived from human bone marrow, through the p38 MAPK pathway [33]. Furthermore, lncRNA HOXA-AS2 was observed to enhance the osteogenesis positive regulation in mesenchymal stem cells by inactivating the NF-κappa B signaling [34]. The above research show the lncRNA participates in the regulation of osteogenic differentiation of MSCs via various pathways, the mechanism of LF-promoting MSCs osteogenic activity has yet to be elucidated. We assume that the contribution of LF in promoting osteogenic differentiation of MSCs may be through affecting the lncRNA expression which may regulate osteogenic differentiation.

Here, we employed a high-throughput RNA sequencing (RNA-seq) approach to explore the expression profiles of lncRNAs in control rBMSCs (CON group) and LF-treated rBMSCs (LF group), with the goal of better understanding the potential role of differential expression of lncRNAs in rBMSCs during osteogenic differentiation by lactoferrin. Herein, our work expands the lncRNAs roles in rBMSCs osteogenic differentiation treated by LF, and to provide new insights into LF development for promoting bone health.

## 2. Results

### 2.1. The Effect of LF on Osteogenic Differentiation Capacity of rBMSCs

**Figure 1:**
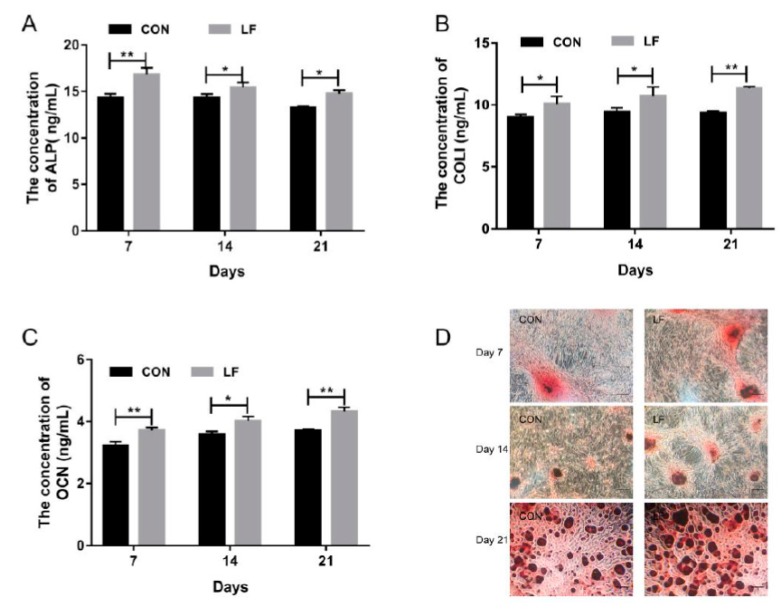
The osteogenic differentiation capacity of control rBMSCs (CON group) and Lactoferrin (LF)-treated rBMSCs (LF group). (**A**) Alkaline phosphatase (ALP) activity of rBMSCs on day 7, 14 and 21; (**B**) COLI expression of rBMSCs on day 7, 14 and 21; (**C**) Osteocalcin (OCN) expression of rBMSCs on day 7, 14 and 21; (**D**) Alizarin Red S staining, magnified images of rBMSCs of LF group and CON group (100×) on day 7, 14 and 21. Data are expressed as mean ± SD of three independent experiments (*n* = 3), the LF group was compared with CON group by unpaired student’s *t*-test (* *p* < 0.05, ** *p* < 0.01).

Osteogenic differentiation of untreated control rBMSCs (CON group) and LF-treated rBMSCs (LF group), were evaluated by examining the early markers of osteogenic differentiation alkaline phosphatase (ALP) level, the later stage marker, osteocalcin (OCN) level, and most abundant protein in bone matrix collagen I (COL I) level on day 7, 14, and 21. The results are presented in Figure 1. The ALP activity of LF group increased significantly (*p* < 0.05) compared with the CON group (Figure 1A). Also, a higher expression level (*p* < 0.05) of COL I in the LF group was shown compared with the CON group (Figure 1B). According to OCN results (Figure 1C), the LF group significantly increased (*p* < 0.05) OCN level compared with the CON group. The calcium deposition, which is a late differentiated marker of osteogenic differentiation, was measured through Alizarin Red S staining. Compared with the CON group, the mineralization increased in the LF group on day 21 (Figure 1D). These results suggest that LF enhanced osteogenic differentiation of rBMSCs, especially on day 14. Therefore, we selected the rBMSCs on day 14 after treatment by LF to analyze the lncRNA profiles by RNA sequencing.

### 2.2. The Effect of LF on lncRNAs Profiles During Osteogenic Differentiation of rBMSCs

**Figure 2:**
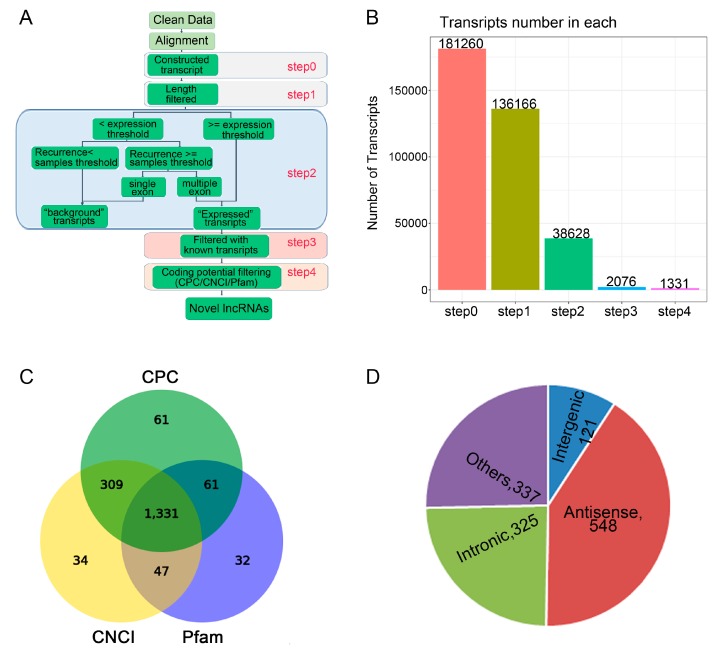
The lncRNAs identified during osteogenic differentiation of CON group and LF group. (**A**) Schematic diagram of the pipeline used for the identification of lncRNAs in rBMSCs. (**B**) The number of lncRNA identification was obtained of four steps. (**C**) Venn diagram showing lncRNAs identified by three types of software. (**D**) Classification of obtained novel lncRNAs according to their genomic positions, distribution of four types of lncRNAs among all 1301 lncRNAs.

We performed identification and profiling of lncRNAs during osteogenic differentiation of CON group and LF groups on day 14 using RNA-seq methodology (Figure 2A). Total RNA was extracted from rBMSCs, and purified from ribosomal RNA, and the fragments were then converted into libraries for high throughput RNA sequencing. After discarding the adaptor and low-quality sequences, raw data consisting of > 152,017,088 clean reads from the Illumina HiSeq 2000 platform were obtained (Table 1). After stringent filtering, clean reads were aligned to the rat reference genome (Available online: ncbi.nlm.nih.gov/genomes: GCF_000001895.5_Rnor_6.0). A highest mapping rate of > 91.17% was in both CON group and LF group (Table 1).

Based on the specific structure and non-coding characteristics of lncRNAs, the transcripts were obtained through 4 steps to identify the annotated and novel lncRNAs (Figure 2B). For identification of lncRNAs, CNCI, CPC, and Pfam software were used and 1331 novel lncRNAs were identified (Figure 2C). According to the relative genomic locations with the coding genes, the identified novel lncRNAs were divided into five classifications (Figure 2D), including 121 of long intergenic lncRNAs or lincRNAs, 325 of intronic lncRNAs, 548 of anti-sense lncRNAs.

### 2.3. Characteristics of lncRNAs and mRNAs

**Figure 3:**
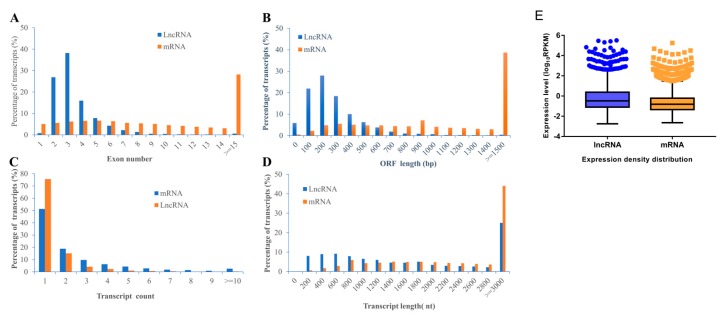
The comparison of features of lncRNAs and mRNAs. (**A**) Exon number. (**B**) ORF length. (**C**) Transcript count. (**D**) Transcript length. (**E**) Expression density distribution. Data are expressed by three independent experiments (*n* = 3).

In our work, in general, we identified a total of 2843 lncRNA (2309 known lncRNAs and 534 novel lncRNAs) and 11,961 mRNA (11,633 known mRNAs and 328 novel mRNAs) in rBMSCs. To characterize the identified lncRNAs, exon number, open reading frame (ORF) length, transcript count, transcript length, and expression density distribution were compared between lncRNAs and mRNAs of rBMSCs (Figure 3). The results revealed a significant differences in the number of exons between lncRNAs and mRNAs; approximately 81.14% of lncRNA transcripts contained 2–4 exons, while only 18.40% of mRNAs transcripts contained 2–4 exons (Figure 3A). Most of mRNAs show longer ORFs length than that of lncRNAs, most lncRNAs (68.31%) consisted of 100 to 300 nt; while about 67.74% of mRNAs consisted ≥ 800 nt (Figure 3B). Furthermore, the distribution of transcripts count showed that most of both lncRNAs (90.74%) and mRNAs (70.14 %) have one or two genes transcripts (Figure 3C). The length of lncRNAs transcripts (55.81%) ranged from 200 to 1600 and most of mRNAs transcripts (44.04%) showed length of ≥ 3000 nt (Figure 3D). These results are in accordance with the findings of previous studies, which concluded that lncRNAs have shorter transcripts and fewer exons than protein-coding genes [35,36,37]. However, lncRNA showed higher expression levels than transcripts of protein coding genes (Figure 3E), which was not consistent with the results of the literature [38].

### 2.4. The Effect of LF on Profiles of Differentially Expressed mRNAs and lncRNAs During Osteogenic Differentiation in rBMSCs

To explore the profiles of differentially expressed mRNAs and lncRNAs during osteogenic differentiation of CON group and LF group, gene expression levels, in terms of transcripts, were quantified by reads per kilo bases per million reads (RPKM) method using HTSeq in Python. Gene expression density distribution (Figure S1A,B) shows that the LF group and CON group were significantly different in genes expression. For principal component analysis (PCA), the loadings for PC1 and PC2 of CON and LF groups (Figure S1C) show that the two groups had a cluster of genes and the gene expression patterns in different samples of each group were highly correlated.

In total, the treatment with LF influenced the expression of 427 genes [Fold change > 1.5 or < −1.5; *p* < 0.05] in rBMSCs on day 14 of osteogenic differentiation (Table S2). Four hundred twenty-seven differentially expressed genes (DEGs), including 120 lncRNAs, 296 mRNA, 8 misc_RNA (Ptger2, LOC108350053, Cyp27a1, LOC102555503, Tmem131, LOC102551633, Dhodh, LOC100362344), and 3 precusor_RNA (Mir3574, Mir3588, Mir29b2), and 3 precusor_RNA (Mir3574, Mir3588, Mir29b2).

**Figure 4:**
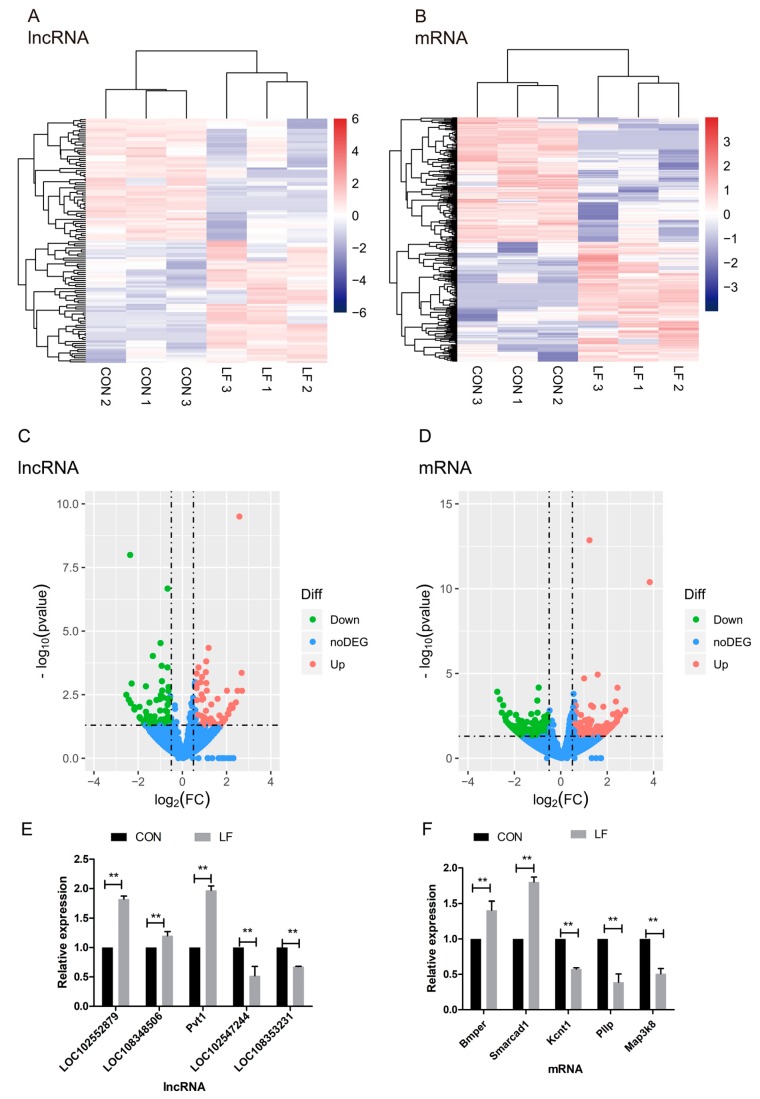
The effect of LF on expression profiles of differentially expressed lncRNAs and mRNAs of rBMSCs during osteogenic differentiation. (**A**,**B**) The unsupervised hierarchical clustering of heatmap show a distinguishable lncRNA or mRNA expression profile between the CON and LF groups. Each row represented one lncRNA or mRNA and each column represented a sample. Red color represented a high relative expression level; blue color represented a low relative expression level. (**C**,**D**) The volcano plot of lncRNA and mRNA expression in CON and LF groups. Each point represented one lncRNA or one mRNA. The red points (up-regulated) and green points (down-regulated) indicated a fold change (FC) in lncRNA or mRNA expression of more than 1.5-fold, and *p* value < 0.05. (**E**) The qRT-PCR results based on the analysis of the five differentially expressed lncRNAs in CON and LF groups. (**F**) The qRT-PCR results based on the analysis of the five differentially expressed mRNAs in CON and LF groups. Data describing the relative fold change are expressed as mean ± SD of three independent experiments (*n* = 3); ** *p* < 0.01.

The differentially expressed LncRNAs (DELs) between CON and LF groups were used to generate a heatmap (Fold change > 1.5 or < −1.5; *p* < 0.05) in unsupervised hierarchical clustering analysis (Figure 4A), the cluster analyses of DELs show two groups could be separate. The heatmap of the differentially expressed mRNAs (DEMs) between CON and LF groups was shown in Figure 4B, which show DEMs in two groups were separated. These selected lncRNA and mRNA expression of rBMSCs during osteogenic differentiation were significantly affected by lactoferrin.

The statistical significance of these differentially expressed lncRNAs, as well as mRNAs in comparison of CON group with LF group (Fold change > 1.5 or < −1.5; *p* < 0.05) were depicted through volcano plots (Figure 4C,D). Additionally, we identified a total of 120 differentially expressed lncRNAs in LF group, compared with CON group (Fold change > 1.5 or < −1.5; *p* < 0.05); sixty of them were upregulated and other 60 were downregulated in rBMSCs, on day 14 of osteogenic differentiation in LF group, compared with CON group (Figure 4C). On the other hand, we identified a total of 296 differentially expressed mRNAs in LF group, compared with CON group (Fold change > 1.5 or < −1.5); one hundred forty-four of them were upregulated and 152 were downregulated in LF group compared with CON group (Figure 4D).

To validate the reliability of the RNA-seq data, we measured expression of the selected five differentially expressed mRNAs and lncRNAs through quantitative real-time PCR (qRT-PCR) validation. In accordance with the RNA-seq data, the two down-regulated lncRNAs were LOC102547244 and LOC108353231, and the three up-regulated lncRNAs were LOC102552879, LOC108348506, and Pvt1 (Figure 4E). However, the three down-regulated mRNAs were Kcnt1, Pllp, and Map3k8; and the two down-regulated mRNAs were Bmper and Smarcad1 (Figure 4F). The results of qRT-PCR are highly consistent with the RNA-seq results.

### 2.5. GO and KEGG Pathway Analysis of DEGs

**Figure 5:**
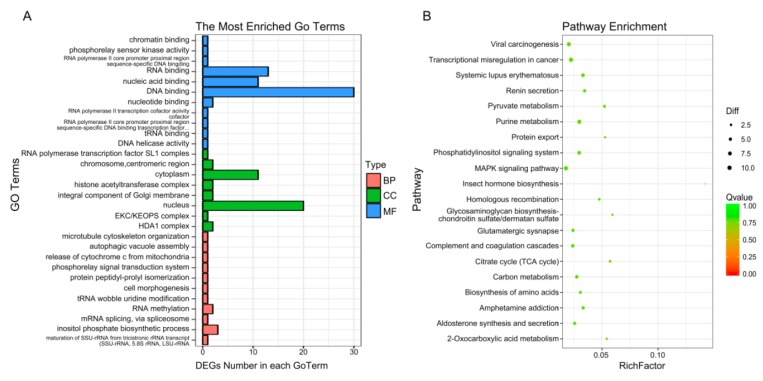
GO and pathway analysis of differentially expressed genes. (**A**) Enriched 30 GO analysis of DEGs. GO analysis covered the three domains biological process, cellular component and molecular function. (**B**) KEGG enrichment analysis of DEGs. The enrichment 30 pathways were shown.

To enrich the significant functions of the differentially expressed genes (DEGs), obtained during osteogenic differentiation of CON and LF groups, GO and KEGG pathway analyses were performed. A total number of 427 DEGs were mainly enriched in categories related to 325 GO functions under biological processes, cellular components, and molecular functions (Table S3), where the most enriched 30 GO terms (Figure 5A) were related to cell morphogenesis, release of cytochrome c from mitochondria, mRNA splicing, via spliceosome and so on.

The KEGG database was used to verify the pathways associated with the possible functions of the DEGs, obtained during osteogenic differentiation of CON and LF groups. Enrich 212 pathways (Table S4), top 30 enriched pathways (Figure 5B), including purine metabolism (nucleotide), pyruvate metabolism (carbohydrate metabolism), phosphatidylinositol signaling system (signal transduction), homologous recombination (replication and repair), carbon metabolism, MAPK signaling pathway (signal transduction), p53 signaling pathway (Cell growth and death), Calcium signaling pathway, and Ras signaling pathway and so on.

### 2.6. Target-gene Prediction of cis- and trans- lncRNA and lncRNA-mRNA Co-expression Networks

To understand how LF contributes to the expression of lncRNAs of rBMSCs during osteogenic differentiation, the potential targets of differentially expressed lncRNAs were predicted via cis- or trans-regulatory manner. For cis action, the genes within the range of 100 kb range of differentially expressed lncRNAs were selected to be the target coding genes, a total of 28 of differentially expressed lncRNAs’ transcripts related to 32 mRNA transcripts within the given range (100 kb) were predicated (Table S5). For trans action, we selected the correlation between lncRNAs and mRNAs with Pearson correlation coefficients not less than 0.8, and over 13 differentially expressed lncRNAs, corresponding to 136 genes for trans regulation, were predicted (Table S6).

**Figure 6:**
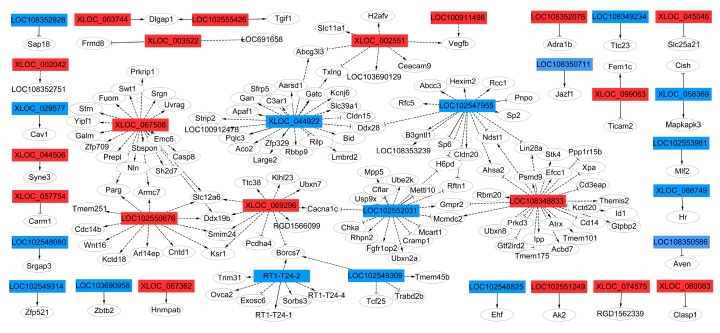
The co-expression network of LncRNA-target genes network. The co-expression network comprised 36 highly conserved lncRNAs and 146 mRNAs. The rectangles represent lncRNA, the circles represent target gene mRNAs. The red represents up regulated lncRNA, and blue represents down regulated lncRNA. The solid line represents cis-regulated targets, the marquee dash line represents trans-regulated targets. The delta target arrow shape represents positive correlation, the T target arrow shape represents negative correlation.

To examine whether these highly conserved lncRNAs and their adjacent protein-coding genes mediate the regulation of osteogenic differentiation of rBMSCs, thirty-six lncRNAs corresponding to 146 mRNAs networks defining the co-expression of genes and lncRNAs and protein coding genes were constructed (Figure 6). The results show that LOC 102550676, XLOC_067508, XLOC_069296, XLOC_044922, LOC 102547955, LOC 102547955, LOC 102547955, LOC 102552031, LOC 108348833, and XLOC_002551 were the key genes regulators, and were correlated with their adjacent genes, specifying an essential important role in the regulation of osteogenic differentiation of LF-treated rBMSCs.

Generally, 146 target genes were predicted as targets of 36 differentially expressed lncRNAs, which were annotated by KEGG database and GO enrich analysis. In addition, 146 genes were classified as known genes, according to the KEGG database, which target genes related to KEGG pathway including: rno05416:Viral myocarditis, rno04210:Apoptosis, rno05152:Tuberculosis, rno05010:Alzheimer’s disease, rno05134:Legionellosis, rno04115:p53 signaling pathway, rno04514:Cell adhesion molecules (CAMs), these pathways may be related to regulation of osteogenic differentiation of rBMSCs (Table S7). The data of GO analysis, related to the osteogenic differentiation of a cellular response to transforming growth factor beta stimulus (TGF-β), positive regulation of glomerular mesangial cell proliferation, regulation of ventricular cardiac muscle cell action potential, SMAD binding, and positive regulation of I-κappa B kinase/NF-κappa B signaling, are shown in Table S8. The results show that the mechanism for enhancement of the osteogenic differentiation of rBMSCs by LF-treatment might be attributed to regulation of lncRNAs expression.

## 3. Discussion

LF is one of the most promising functional food ingredients present in milk, with many biological functions [39], especially for LF-promoting stem cell proliferation and osteogenic differentiation, and in protecting against apoptosis [17,40,41,42], attracting interest from bone health scientists and researchers. Therefore, understanding the underlying mechanism for the enhancement of osteogenic differentiation of BMSCs, induced by LF, will contribute to its application in food and therapeutic industries. In our present study, we investigated whether LF has a potential effect on lncRNAs expression profiles during the osteogenic differentiation of rBMSCs. To clarify the effect of LF on lncRNA expression profiles, we performed lncRNA expression profiles by RNA-seq through Illumina HiseqTM 2000 high throughput sequencer platform. The results show that lactoferrin affected the lncRNAs expression during osteogenic differentiation in rBMSCs, thus the mechanism of osteotropic activity of LF referred to the lncRNA regulation. To the best of our knowledge, this is the first study that has investigated the changes in profile expression of lncRNAs, during osteogenic differentiation of LF-treated rBMSCs culture, and explored the underlying mechanism of rBMSCs for promoting osteogenic activity induced by LF via RNA-seq.

In our study, LF at concentrations of 100 µg/mL was used for treatment of rBMSCs because this concentration was found to significantly promote proliferation in our previous study [43]. In some studies, 100 μg/mL LF has the best effect on promoting osteoblast proliferation and osteogenic differentiation [16,20]. In addition, 100 μg/mL LF also significantly stimulates proliferation and osteogenic differentiation of human adipose stem cells [17] and C3H10T1/2 Mesenchymal Stem Cells [18]. The alkaline phosphatase (ALP) is the early marker of osteogenic differentiation [44]. LF significantly enhances ALP expression of C3H10T1/2 mesenchymal stem cells on day 7 of osteogenic differentiation [18], and significantly promotes the ALP expression of MEG63 human osteoblast-like cells on day 14 of osteogenic differentiation [45]. The results of these studies were consistent with our results (Figure 1A). However, in Montesi et al. studies, 10 μg/mL LF decreases ALP level of MSCs on day 14 of osteogenic differentiation, compared with the control group [40], which is inconsistent with our research. This inconsistency may be attributed to the different cell origins, LF concentrations, and ALP assay methods used in our study. COLI and OCN are maturation state marker or transcription factors of osteogenic differentiation. OCN is involved in controlling the mineralisation process, which appeared at a late stage of osteogenic differentiation. Zou et al. found that OCN expression has the highest expression level on day 21 during osteogenic differentiation [44], which is similar with our results (Figure 1C). Takayama et al. showed that LF has a promoting effect on calcium deposition of MEG63 cells on day 21 of osteogenic differentiation [45], which is consistent with our results (Figure 1D). The promoting of osteogenic differentiation of rBMSCs by LF treatment was verified via analysis of ALP, COLI, OCN expression, and the calcium deposition during osteogenic differentiation of rBMSCs on day 7, 14, and 21. The obtained results reveal that lactoferrin enhanced the osteogenic differentiation of rBMSCs apparently. In our study, LF significantly increased the related osteogenic differentiation marker expression on day 14 of osteogenic differentiation of rBMSCs, which coincided with the report of Jiang et al. [46], thus, differential expression profiles of lncRNAs and mRNAs in rBMSCs, and the effect of LF on day 14, were studied by RNA-seq.

Our study is the first lncRNA profiling analysis of rBMSCs induced by untreated and treated-LF during osteogenic differentiation on day 14 by high throughput sequencing. In general, a total of 2843 lncRNA (2309 known lncRNAs and 534 novel lncRNAs) and 11,961 mRNA (11,633 known mRNAs and 328 novel mRNAs) were identified, in comparison with mRNAs, the lncRNA transcripts contained fewer exons, show shorter overall transcripts length and ORF length, which was in agreement with previous reports from others studies [35,36,37]. Moreover, compared with mRNAs, the lncRNAs have a higher expression level. This is inconsistent with other studies, which may be due to the reads per kilo bases per million reads (RPKM), used to evaluate the expression of transcripts in our study, not fragments per kilo-base of exon per million fragments mapped (FPKM). In short, our study firstly exhibits the feature of mRNA and lncRNA during osteogenic differentiation of rBMSCs, treated by LF, and shows that the LF-inducing osteogenic activity not only existed through the regulation of transcriptional level, but might also be related to the participation in of lncRNA in post-transcriptional and post-translational levels.

Mechanism of MSCs osteogenic differentiation is regulated by complex pathways at transcriptional, post-transcriptional and post-translational levels [42,47,48,49,50], and the related osteogenesis signaling pathways, including TGF-β signaling [21], Wnt signaling [51], Hedgehog [52], Notch [53], NF-κappa B signaling [5], Ras [54], and Calcium Signaling [55]. In our study, a total number of 427 differential genes were identified and found to be related to osteogenic development pathways, such as MAPK signaling pathway, p53 signaling pathway (Cell growth and death), calcium signaling pathway and so on. Evidences have shown that LF have a capacity of modulating bone formation and bone resorption, LF can regulate osteoblast differentiation through PKA and p38 MAPK pathways by lactoferrin’s Receptor LRP1 [16,56], and promote cell proliferation via MAPK signaling pathways [20]. Also, the MAPK signaling pathway have been demonstrated be associated with osteogenic differentiation of human bone marrow mesenchymal stem cells [57]. In this study, the JNK/p38 MAPK pathway (downregulated gene, including IL1R, Cdc42, Map3k8, upregulated gene, including Dusp7, Cacna1c) is suggested to be involved in the effect of LF on rBMSCs osteogenic differentiation. In particular, p53 was found to suppress the expression of Runx2 [58] and Osx [59], and in modulating osteoblast differentiation [60]. In our results, p53 (downregulated gene, including cd82, Pmaip1, Shisa8, Zfp385c) is shown to be involved in osteogenic differentiation of LF-treated rBMSCs. In addition, the mRNA expression of calcium channels (cacana1c, cacana1e, cana1g and cacna1i) were activated during osteogenic differentiation [61]. Meanwhile, the calcium signaling pathway was affected in the osteogenic differentiation of mesenchymal stem cells [62]. In our work, calcium signaling pathway regulation genes, including Bdkrb1, Cacna1c, Rcn1, Cabp4, and Itpkc were upregulated, and genes, including Tmem45b, Cxcl17, were downregulated. Collectively, these results show that enhancement of osteogenic differentiation of rBMSCs by LF may be associated to the MAPK signaling pathway, p53 signaling pathway, and calcium signaling pathway, which pathways may be regulated by the different expression mRNA and lncRNAs. However, the effect of LF on lncRNA expression profiling of rBMSCs, during osteogenic differentiation, is not reported. Our results show that LF contributed to altering the lncRNA and mRNA expression, which participated in the osteogenic differentiation of rBMSCs.

The lncRNAs are long noncoding RNA that regulate the functions of genes and proteins through various mechanisms, including the modulation of in epigenetic, transcriptional, and post-transcriptional gene and other specific regulation modes [22,23,28]. Previous studies have demonstrated that lncRNA regulates osteogenic differentiation of osteoblast and MSCs [63,64,65,66,67]. In this study, LF affected lncRNA profiles of rBMSCs during osteogenic differentiation through 60 upregulated and downregulated lncRNAs (*p* < 0.05, Fold change > 1.5 or < 1.5). Since lncRNAs are able to modulate the expression of protein coding genes in cis or trans [68], among the dysregulated lncRNA transcripts, we predicted the target gene according to the position of the differentially expressed lncRNAs, and their relationship with mRNAs via a cis- or trans-regulatory manner. In total, 36 lncRNAs corresponding to 146 mRNAs networks describing the co-expression of genes and lncRNAs were identified, indicating a potentially important role in regulating the changes in genes expression in LF-treated rBMSCs. The GO and pathway results of DELs target genes suggested a possible involvement of DELs in a variety of biologicals processed, such as a cellular response to transforming growth factor beta stimulus (TGF-beta signaling pathway), co-SMAD binding, and I-κappa B kinase/NF-κappa B signaling, which are likely to be related to osteogenic differentiation or bone formation. Zhang et al. [69] reported that, during osteogenic differentiation of human bone marrow mesenchymal stem cells, the lncRNA may participate in the regulation of MAPK signaling pathway and TGF-beta signaling pathway, which are associated with osteogenic differentiation. Moreover, TGF-beta signaling pathway was related to adipose-derived mesenchymal stem cells osteogenesis and bone formation of miR-10b via target gene Smad2 [70]. In our results, lncRNA target gene related to cellular response to transforming growth factor beta stimulus (TGF-β) (target gene Cav1, Id1, Usp9x, Apaf1) and co-SMAD binding (target gene, Usp9x, Tgif1). Usp9x is an important osteogenic regulating protein, which plays a major role in activating BMP signaling [71]. Therefore, the lncRNA LOC102552031 may participate in regulation of the osteogenic differentiation of LF-treated rBMSCs via target Usp9x in trans. In positive regulation of I-κappaB kinase/NF-κappaB signaling pathway (target gene Cflar, Casp8, Ticam2, Tmem101), Cflar and Casp8 play a critical role in autophagy, necroptosis, and apoptosis [72]. Therefore, LOC102552031 and XLOC_067508 lncRNAs may regulate the apoptosis process of rBMSCs via target gene Cflar and Casp8 in trans. The obtained results indicate that lncRNAs have a potentially important role in the regulation of LF-treated rBMSCs differentiation. However, the precisely regulated function of lncRNA in osteogenic induced by LF will be further verified by other manners. Herein, we concluded that LF, affected lncRNAs and mRNAs expression, which contributed to the osteogenic differentiation of rBMSCs via complex signaling pathway. Generally, the function of lncRNAs is reflected by their effects on adjacent gene protein-coding genes via cis and trans manner.

These results may help understand the role of lncRNAs in the enhancement of the osteogenic differentiation of MSCs by LF. However, the mechanism of regulation during osteogenic differentiation of rBMSCs, by differentially expressed lncRNAs function, is still unclear and warrants further investigation.

## 4. Materials and Methods

### 4.1. Cell Culture

Primary Sprague Dawley (SD) rat bone mesenchymal stem cells (rBMSCs) were purchased from the Cell Bank of Typical Culture Preservation Committee of Chinese Academy of Science (Shanghai, China). The cells were cultivated in Dulbecco’s Modified Eagle Medium (DMEM; Gibco life technologies Co. Ltd. Carlsbad, CA, USA) containing 10% fetal bovine serum (FBS, Gemini Bio-Products, West Sacramento, CA, USA), 100 U/mL penicillin and 100 mg/mL streptomycin, at 37 °C in 5% CO_2_. After reaching 80% confluency, rBMSCs were passaged using 0.25% trypsin-EDTA (Gibco life technologies Co. Ltd. Carlsbad, CA, USA). Cells at passage 3 and 4 were used in the experiments.

### 4.2. Osteogenic Differentiation of rBMSCs

For the osteogenic differentiation assays, rBMSCs were seeded into a 6-well tissue plate, at a cell density of 1 × 10^6^ cells/well, and cultured in osteogenic differentiation medium (Cyagen Biosciences Inc. Guangzhou, China) with (100 μg/mL) or without bovine lactoferrrin (LF, 96.3% of purity, Westland Co-operative Dairy Co. Ltd., Hokitika, New Zealand, and the iron saturation of bovine lactoferrin was 15%) for 7, 14, and 21 days and the medium renewed every 3 days of the culture. The cells were then rinsed twice with phosphate buffered saline (PBS) (Solarbio Science and Technology Co. Ltd. Beijing, China). Markers of osteogenic differentiation-alkaline phosphatase (ALP), collagen I (COL I), osteocalcin (OCN) secretion were detected in cell culture supernatants with an enzyme-linked immune-sorbent assay (ELISA) kit (Jianglai Bio, Shanghai, China) following the product manufacturer’s instructions and measured at a wavelength of 450 nm by multifunctional microplate reader scanning (Infinite M200PRO, TECAN, Männedorf, Switzerland).

### 4.3. Alizarin Red Staining of rBMSCs

Alizarin red staining performed in the extracellular matrix mineralization of osteogenic differentiation of rBMSCs in cultured with, or without, 100 μg/mL LF for a period of 7, 14, and 21 days. Briefly, rBMSCs were rinsed twice with PBS, followed by fixed for 30 min in 4% paraformaldehyde. After rinsing the cells twice using PBS, rBMSCs were stained using 1% alizarin red S for 15 min at room temperature and observed under the microscope (JEM-1200EX, Hitachi, Tokyo, Japan).

### 4.4. RNA Extraction, Library Construction, and High-throughput RNA Sequencing (RNA-Seq)

The total RNA was extracted through TRIzol reagent (Invitrogen Corp., Carlsbad, CA, USA) according to the manufacturer’s instructions and their integrity was examined. Quantitative analysis of total RNA, using an RNA 6000 Nano Kit (Agilent Technologies, Palo Alto, CA, USA), by an Agilent 2100 Bio analyzer (Bioanalyzer 2100 system) was conducted.

To ensure accurate lncRNA analysis, ribosomal RNA was removed from the RNA through Ribominus kit (Life Technologies, Foster City, CA, USA). RNA samples from three rBMSCs groups with RNA Integrity Number (RNI) ≥ 8 were subjected to library construction and high-throughput RNA sequencing (RNA-seq) at Beijing Genome Institute (BGI, Beijing, China), using an Illumina HiSeq™ 2000 high throughput sequencer platform with 100 paired end sequencing, according to the manufacturer’ specifications.

Low-quality reads were removed, and the raw reads trimmed and mapped against the rat reference genome in NCBI (Available online: ncbi.nlm.nih.gov/genomes: GCF_000001895.5_Rnor_6.0) with HISAT (hierarchical indexing for spliced alignment of transcripts) version 0.1.6 [73]. The mapped reads were assembled into contigs using StringTie version 1.0.4.4.1 [74] and Cufflinks version 2.2.1 [75].

### 4.5. LncRNAs Identification and Classification

To identify the putative novel lncRNAs in rBMSCs, the mapped reads (of each sample in rBMSCs) were assembled using the software Cufflinks version 2.2.1 and StringTie version 1.0.4 (in a reference-based approach) [74]. Cufflinks was also used for comparison of candidate sequences with the recognized lncRNAs [75]. The following procedure was used to select and identify putative novel lncRNAs: (1) transcripts length ≥ 200 bp were removed; (2) transcripts with multiple exons (transcript exon > 2/the number of exons > 1) and RPKM ≥ 2 were used; (3) transcripts overlap with known lncRNA on the opposite strand and the precursor transcript of mRNA were filted; (4) and transcripts predicted to be lncRNA were obtained by coding potential calculator (CPC) version v0.9-r2 [76], coding non coding index (CNCI) version 2.1 [77], and Pfamscan (Pfam) version 3.0 [78]. The obtained lncRNAs were classified according to their reference-based locations in the genome.

### 4.6. Differential Expression Genes Analysis of Transcripts

After mapping to the rat reference genome, the genes expression levels, related to the transcripts, were further quantified through the reads per kilo bases per million reads (RPKM) method, using HTSeq version 0.6.1 in Python [79]. Differentially expressed genes (DEG) of coding mRNAs and lncRNAs were detected through DEGseq2 version 1.4.5 [80]. Differentially expressed lncRNAs and mRNAs with statistical significance threshold of *p*-value < 0.05 and fold changes above 1.5 were assigned as being differentially expressed.

### 4.7. Bioinformatics Analysis

Pathway analysis for the differentially expressed mRNAs was conducted through the Kyoto Encyclopedia of Genes and Genomes (KEGG) pathways. The Gene Ontology (GO) enrichment analysis of differentially expressed genes was performed using GOseq version 1.16.2 of R package (http://bioconductor.org/packages/release/bioc/html/goseq.html).

### 4.8. Target-Gene Prediction

Since lncRNA can cis- and trans-regulate target genes, protein-coding genes, found within 100 kb locations of chromosomes from the lncRNA, were screened out using R package and designated as lncRNA potential cis- regulated targets.

To investigate the trans- type interface, the Pearson’s correlations coefficients (*r* > 0.8 or *r* < −0.8) among lncRNA and protein-coding genes were examined. Cytoscape software (version 3.7.0; available online: www.cytoscape.org) was used to accomplish the co-expression-network analysis, and to determine the correlated expression of genes.

### 4.9. Quantitative Real-Time PCR Validation

Randomly chosen DEGs were used for the validation of RNA-seq results by qRT-PCR. Primer 6.0 software was used to design the gene-specific primers and synthesized by Sangon Biotech Company (Shanghai, China). The expression patterns of the chosen lncRNA and mRNAs were studied through qRT-PCR using RealmasterMix (SYBR Green, TIANGEN, China) following the manufacturer’s instructions. Gapdh mRNA was used as an internal control. The method 2^−ΔΔ*C*t^ method was used for the quantitative results to interpret the fold changes, followed by the statistical analysis. All the designed primer sequences are given in Table S1.

### 4.10. Statistical Analysis

All of our experiments were performed in three independent replicates (*n* = 3), and data are presented as mean ± SD. Unpaired students’ *t*-tests was performed to evaluate the differences between the CON group and LF group. Statistical significance was set at *p* < 0.05.

## 5. Conclusions

In conclusion, our study firstly investigated LF affected by the lncRNAs expression profiles during osteogenic differentiation of rBMSCs. These differentially expressed lncRNAs (60 up-regulated and 60 down-regulated) may contribute to the osteogenic differentiation of LF-treated rBMSCs via regulation of TGF-β and I-κappa B kinase/NF-κappa B signaling pathways. These results reveal LF-promoting osteogenic differentiation of rBMSCs was a complex process, not only by regulation of mRNAs, but also by regulation of lncRNAs expression. This study provides an experimental basis for further research regarding mechanisms that explain the effect of LF on osteogenic differentiation of MSCs.

## Figures and Tables

**Table 1:** RNA-Seq read mapping summary.

Sample	Total Reads	Total Mapped Reads	Unique Mapped Reads	Reads Mapped in Paired	Detected Gene Number	Detected SNP Number	Detected InDel Number
CON 1	152,017,088	94.17%	75.18%	92.02%	9793	50,655	8142
CON 2	151,919,042	93.78%	74.16%	91.80%	9485	46,504	7442
CON 3	152,842,644	96.66%	78.92%	94.02%	9777	50,140	8047
LF 1	151,975,658	96.84%	77.27%	94.59%	9890	51,318	8190
LF 2	152,415,436	95.30%	73.01%	92.95%	9171	43,676	7003
LF 3	151,898,670	91.47%	65.73%	88.37%	9219	42,587	6739

## Supplementary Materials

Supplementary materials can be found at https://www.mdpi.com/1422-0067/20/19/4834/s1.

## Abbreviations

ALP	Alkaline phosphatase
BMSCs	Bone marrow mesenchymal stem cells
CNCI	Coding non coding index
COL I	Collagen type I
CPC	Coding potential Calculator
DEG	Differentially expressed gene
GO	Gene ontology
KEGG	Kyoto encyclopedia of genes and genomes.
LF	Lactoferrin
lncRNAs	Long non-coding RNAs
MAPK	Mitogen-activated protein kinase
ncRNAs	Non-coding RNAs
OCN	Osteocalcin
Pfam	Pfamscan
RNA-seq	RNA Sequencing
RPKM	Reads per kilo bases per million reads
