# Supplementary material for: Effect of Lactoferrin on the Expression Profiles of Long Non-coding RNA during Osteogenic Differentiation of Bone Marrow Mesenchymal Stem Cells
Source: Int J Mol Sci. 2019 Sep 28;20(19):4834. doi: 10.3390/ijms20194834 (PMC6801644; doi:10.3390/ijms20194834)
Supplement: Supplementary file 1 [file ijms-20-04834-s001.zip › ijms-588882-SI/Supplement/Additional file 2 Figure Supplement 1.docx]

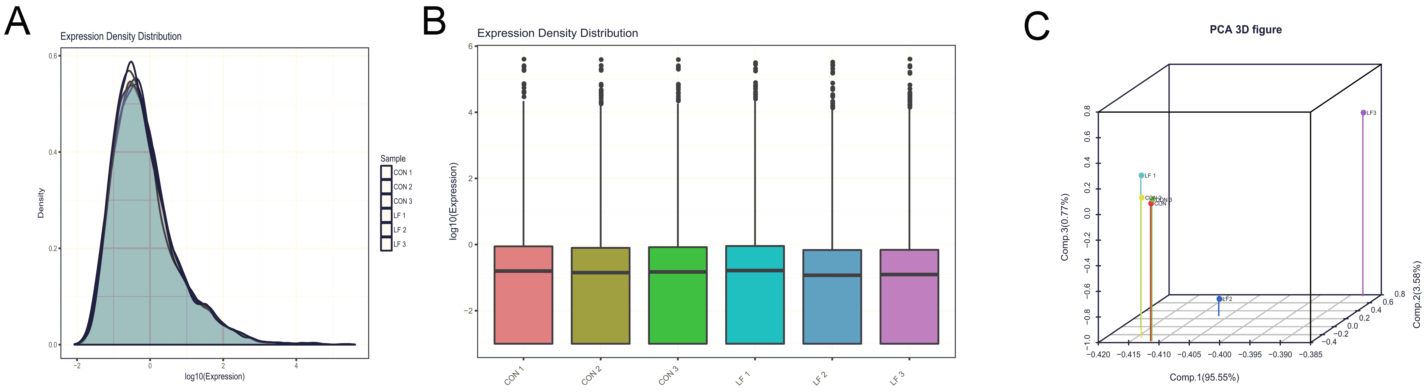


Figure S1 The gene expression distribution of control rBMSCs (CON group) and LF-treated rBMSCs (LF group). (A) The gene expression density distribution of CON and LF. The gene expression of rBMSCs were quantified by reads per kilo bases per million reads (RPKM) method using HTSeq in Python. (B) The box plot of expression density distribution of CON and LF. One box represents one sample. (C) The principal component analysis (PCA) 3D figure. One circle ponit represent a sample, it show that two groups had a cluster of genes.
