# Supplementary figures and images for: Effect of Lactoferrin on the Expression Profiles of Long Non-coding RNA during Osteogenic Differentiation of Bone Marrow Mesenchymal Stem Cells
Source: Int J Mol Sci. 2019 Sep 28;20(19):4834. doi: 10.3390/ijms20194834 (PMC6801644; doi:10.3390/ijms20194834)

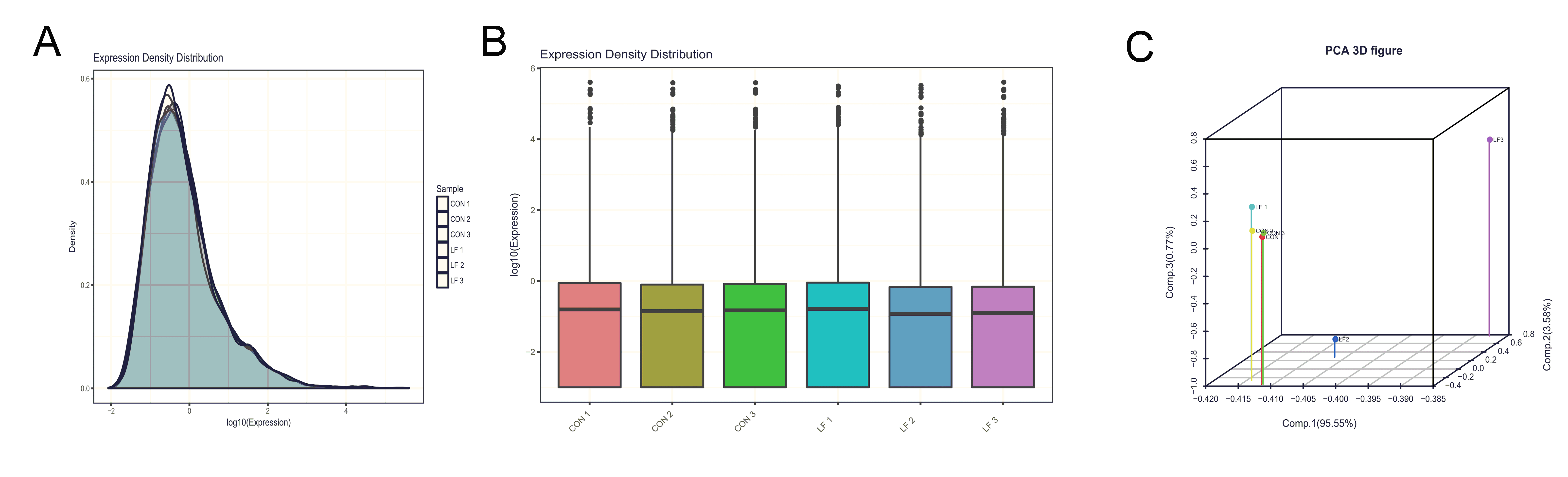

Supplement: Supplementary file 1 [file ijms-20-04834-s001.zip › ijms-588882-SI/Supplement/Additional file 2 Figure Supplement 1.tif]
